# Fast Quasi-Centroid Molecular Dynamics for Water and Ice

**DOI:** 10.1021/acs.jpcb.3c05028

**Published:** 2023-10-13

**Authors:** Joseph E. Lawrence, Annina Z. Lieberherr, Theo Fletcher, David E. Manolopoulos

**Affiliations:** † Laboratory of Physical Chemistry, ETH Zürich, 8093 Zürich, Switzerland; ‡ Physical and Theoretical Chemistry Laboratory, Department of Chemistry, University of Oxford, South Parks Road, Oxford OX1 3QZ, United Kingdom

## Abstract

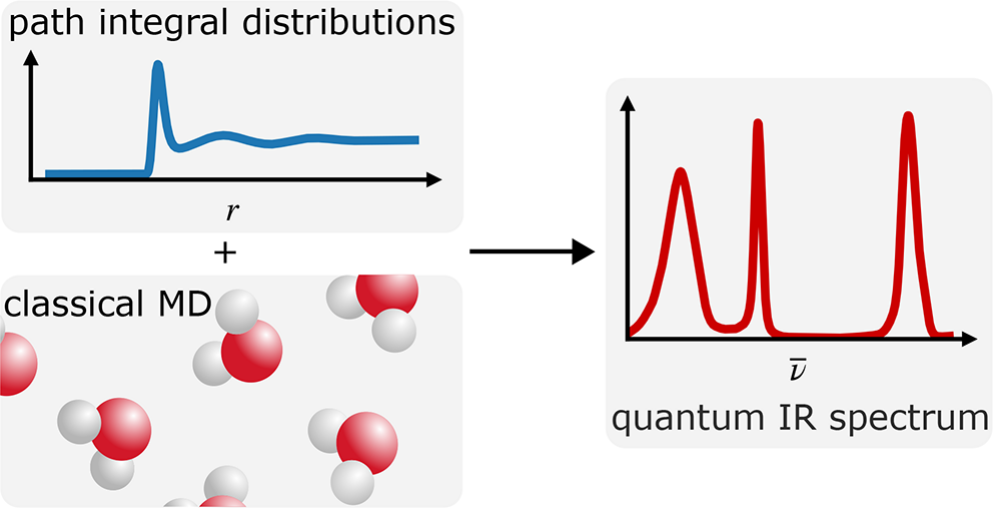

We describe how the fast quasi-centroid molecular dynamics (f-QCMD) method can be applied to condensed-phase systems by approximating the quasi-centroid potential of mean force as a sum of inter- and intramolecular corrections to the classical interaction potential. The corrections are found by using a regularized iterative Boltzmann inversion procedure to recover the inter- and intramolecular quasi-centroid distribution functions obtained from a path integral molecular dynamics simulation. The resulting methodology is found to give good agreement with a previously published QCMD dipole absorption spectrum for liquid water and satisfactory agreement for ice. It also gives good agreement with spectra from a recent implementation of CMD that uses a precomputed elevated temperature potential of mean force. Modern centroid molecular dynamics methods, therefore, appear to be reaching a consensus regarding the impact of nuclear quantum effects on the vibrational spectra of water and ice.

## Introduction

The importance of experimental vibrational spectra as a probe of inter- and intramolecular interactions has generated considerable interest in the development of methods for their simulation. For small gas-phase molecules, it is possible to compute vibrational spectra exactly using wave function methods. However, for condensed-phase systems such as liquid water and ice, it is not feasible to solve the Schrödinger equation exactly due to the exponential scaling of quantum mechanics with dimensionality. It is nevertheless possible to compute the static equilibrium properties of condensed-phase systems using imaginary-time path-integral techniques, which are based on the isomorphism between distinguishable quantum particles and classical ring polymers.^1^ Indeed, the path integral molecular dynamics (PIMD) method has been used for almost 40 years^2^ to shed light on quantum mechanical (zero point energy and tunneling) effects in condensed phases. There has, therefore, been particular interest in adapting methods based on imaginary time path integrals to provide approximations of the dynamical properties of condensed-phase systems, such as their vibrational spectra.

In a pioneering series of papers published almost 30 years ago, Cao and Voth focused on the centroid of the ring polymer and showed how this could be used to develop such a dynamical approximation.^3−6^ Their centroid molecular dynamics (CMD) method is simply classical molecular dynamics on an effective potential: the potential of mean force obtained by averaging the thermal fluctuations of the ring polymer around its Cartesian centroid. Various approximations to this effective potential had been suggested previously,^7,8^ but to keep their method consistent with static equilibrium properties, Cao and Voth chose not to make any approximation to the quantum mechanical partition function.^3^ Instead, they developed an adiabatic algorithm that enabled them to calculate the exact mean force on the centroid “on the fly” during the course of a PIMD simulation.^6^ This adiabatic algorithm has since been used to apply CMD to a wide range of systems and to study the impact of nuclear quantum effects on a variety of dynamical observables, including vibrational spectra.^9,10^

The resulting vibrational spectra are generally quite reliable at high temperatures, and the method is expected to give a good approximation to the quantum transition frequency ω_10_ = (*E*_1_ – *E*_0_)/ℏ of a one-dimensional anharmonic oscillator in the low-temperature limit.^11^ However, CMD has since been found to suffer from a “curvature problem” in multidimensional systems that possess both stretching and angular (bending, torsional, or librational) modes.^12^ The ring polymer spreads out around the angular coordinates as the temperature is lowered, causing a softening of the centroid potential of mean force in the radial directions that results in a spurious red-shifting and broadening of the stretching bands in the CMD spectrum.^12^ While the shifting and broadening of the O–H stretching band are not especially pronounced in liquid water at room temperature,^10,13^ the problem becomes more severe as the temperature is lowered, and it is certainly quite noticeable in ice at 150 K.^14^

To solve the curvature problem, Althorpe and coworkers have introduced a “quasi-centroid” molecular dynamics (QCMD) method in which the dynamical variable is the quasi-centroid of the ring polymer rather than its centroid.^15^ This quasi-centroid is defined in terms of certain radial and angular coordinates that are specific to the problem at hand. For example, the quasi-centroid O–H bond lengths of a water molecule are
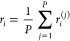
1and the quasi-centroid H–O–H bond angle is
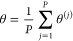
2where *r*_1_^(*j*)^ and *r*_2_^(*j*)^ are the two O–H bond lengths and θ^(*j*)^ is the H–O–H bond angle of the molecule in the *j*-th bead of the ring polymer necklace. Replacing the centroid with the quasi-centroid solves the curvature problem because the radial quasi-centroids *r*_*i*_ remain at realistic bond lengths when the temperature is lowered, and the bead distributions in the rotational and bending coordinates become less compact.

Althorpe and coworkers have shown that QCMD vibrational spectra have physically reasonable stretching bands at both high and low temperatures for systems including gas-phase water^15^ and ammonia^16^ and condensed-phase water and ice.^14^ These QCMD calculations were performed using a quasi-centroid adaptation^15^ of Cao and Voth’s adiabatic CMD algorithm.^6^ Unfortunately, this is even more computationally expensive than the original adiabatic CMD algorithm. One has to use a small time step to correctly integrate the equations of motion of the adiabatically separated ring polymer internal modes, and the fact that these modes are coupled to the quasi-centroid leads to additional complications that have to be dealt with rather carefully.^17^

To avoid these difficulties, we have recently developed a “fast” implementation of QCMD in which one precomputes an approximation to the quasi-centroid potential of mean force that is consistent with the quasi-centroid distribution functions obtained from a PIMD simulation.^18^ The idea here is similar to that of an earlier “fast CMD” method of Hone, Izvekov, and Voth,^19^ and in both cases, the potential of mean force is calculated by adding a small correction to the classical interaction potential. However, while Hone et al. used force matching to fit their correction to PIMD forces, we have found it more convenient in the quasi-centroid case to use iterative Boltzmann inversion^20,21^ (IBI) to fit the correction to PIMD distribution functions. The resulting methodology reproduces Althorpe and coworker’s QCMD results for gas-phase water and ammonia, and it enabled us to perform the first QCMD calculations for gas-phase methane.^18^ In Section 2, we will describe how fast QCMD can be generalized from the gas phase to the condensed phase and used to simulate the vibrational spectra of liquid water and ice.

More recently still, Kapil and coworkers have developed an alternative method in which the centroid is retained in preference to the quasi-centroid, but the centroid potential of mean force is calculated at an elevated temperature.^22^ This is chosen to be sufficiently high that the curvature problem has not yet become an issue and yet sufficiently low that the high-frequency intramolecular stretching vibrations are already in their quantum ground states. A suitable temperature for liquid water can be found by monitoring the convergence of the PIMD distribution function in the O–H stretching coordinate as the temperature is lowered. Since this stops changing at around 600 K, where the curvature problem is not yet an issue, a centroid potential of mean force constructed at this temperature can be used in simulations of the vibrational spectrum at lower temperatures. Kapil and coworkers employed machine learning techniques to construct the 600 K centroid potential of mean force and demonstrated that the resulting ‘elevated temperature path integral ground state’ (Te PIGS) method avoids the curvature problem in liquid water at 300 K and in ice at lower temperatures.^22^ We shall compare our f-QCMD results with theirs for both of these systems in Section 3.

## Fast QCMD

A fast QCMD simulation of a system such as liquid water or ice has three stages: (i) a short PIMD simulation is used to construct quasi-centroid distribution functions at the target temperature and density, (ii) an IBI (coarse-graining) procedure is used to fit these distribution functions to those of an effective classical potential of mean force, and (iii) a classical simulation of the vibrational spectrum is performed on the resulting quasi-centroid potential of mean force. We now describe each of these stages in turn.

### Quasi-Centroid Distribution Functions

We have already defined what we mean by the quasi-centroid bond lengths and the quasi-centroid bond angle of a given water molecule at a particular configuration in a *P*-bead PIMD simulation [eqs 1 and 2]. These three coordinates, *r*_1_, *r*_2_, and θ, suffice to determine the size and shape of the quasi-centroid water molecule but not its position or orientation in space. In order to calculate intermolecular quasi-centroid distribution functions, we need to know the actual positions ***r***_X_ of the quasi-centroids of all three atoms (X = O, H_1_, and H_2_) in the molecule. To determine them, we follow Althorpe and coworkers in setting the center-of-mass of the quasi-centroid molecule to be that of the Cartesian centroid molecule and choosing the orientation of the quasi-centroid molecule to be as close as possible to that of the Cartesian centroid molecule.^15,17^ Given an arbitrarily aligned and positioned quasi-centroid molecule constructed from *r*_1_, *r*_2_, and θ, we first find the rotation matrix **U** that minimizes the sum of mass-weighted squared deviations^23^
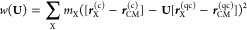
3where ***r***_X_^(c)^ and ***r***_X_^(qc)^ are the centroid and quasi-centroid of atom X and ***r***_CM_^(c)^ and ***r***_CM_^(qc)^ are the corresponding molecular centers of mass, respectively. The translated and rotated quasi-centroid coordinates are then
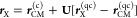
4This procedure is easier than it may seem because there exists a simple and efficient algorithm for finding the optimum **U** in eq 3 and performing the rotation in eq 4, as described in ref (23). Applying this algorithm to each water molecule, in turn, rapidly generates a set of quasi-centroid coordinates {***r***_X*I*_} for each atom X within each molecule *I* in any given configuration of the PIMD simulation.

Armed with these quasi-centroid coordinates, it is straightforward to calculate intermolecular quasi-centroid radial distribution functions from the configurations visited in the PIMD simulation. These radial distribution functions are defined as
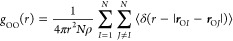
5a
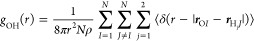
5b
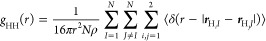
5cwhere the angular brackets denote averages over the path integral configurations that are used to generate the quasi-centroid coordinates ***r***_X*I*_, and ρ = *N*/*V* is the bulk number density of water molecules. All three radial distribution functions are straightforward to accumulate as histograms during a short PIMD simulation.

To complete the information that is used to construct our approximation to the quasi-centroid potential of mean force, we use the same form for the intramolecular distribution functions as we used in our fast QCMD study of the water monomer.^18^ These are
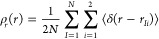
6and
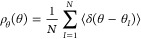
7where *r*_*Ii*_ and θ_*I*_ are the path integral bead averages of the intramolecular O–H_*i*_ distance and the intramolecular H–O–H bond angle in molecule *I* [see eqs 1 and 2], respectively. These distribution functions can be accumulated as histograms during the same PIMD simulation used to calculate *g*_OO_(*r*), *g*_OH_(*r*), and *g*_HH_(*r*).

### Quasi-Centroid Potential of Mean Force

The central observation behind fast QCMD is that, by definition, the quasi-centroid distribution functions in eqs 5, 6, and 7 will be the same whether the angular brackets ⟨·⟩ indicate an average over a classical NVT simulation on the quasi-centroid potential of mean force or over a PIMD NVT simulation in which the locations of the quasi-centroids ***r***_X*I*_ are calculated from the path-integral beads. This equivalence allows us to use standard coarse-graining techniques such as IBI to construct an approximation to the quasi-centroid potential of mean force that is consistent with the distribution functions obtained from the PIMD simulation.^18^

To do this, we begin as we did for gas-phase water by writing the quasi-centroid potential of mean force as a correction to the classical interaction potential, but now with inter- as well as intramolecular correction terms
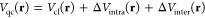
8Here, **r** is a configuration vector of the entire quasi-centroid system, *V*_cl_(**r**) is the classical interaction potential, and Δ*V*_intra_(**r**) and Δ*V*_inter_(**r**) are the intra- and intermolecular correction terms, respectively. For computational expedience, both in the IBI and in the subsequent molecular simulation on the potential of mean force *V*_qc_(**r**), we approximate Δ*V*_intra_(**r**) as we did in our study of gas-phase water^18^
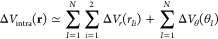
9and Δ*V*_inter_(**r**) as a sum of pairwise contributions
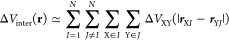
10

### Iterative Boltzmann Inversion

The corrections in eqs 9 and 10 can be found by IBI.^20,21^ While there are alternative methods that could be applied to this problem,^24,25^ we have found IBI to be the most efficient and robust. The algorithm can be explained as follows. One begins by setting the initial corrections Δ*V*_intra_^(0)^(**r**) and Δ*V*_inter_^(0)^(**r**) to zero, such that the initial guess for quasi-centroid potential is just the classical potential *V*_cl_(**r**). In the *i*th iteration of the algorithm, the system is propagated on the effective potential *V*_qc_^(*i*)^(**r**) obtained by adding potential corrections Δ*V*_intra_^(*i*)^(**r**) and Δ*V*_inter_^(*i*)^(**r**) to *V*_cl_(**r**). The corresponding approximations to the distribution functions ρ_*r*_^(*i*)^(*r*), ρ_θ_^(*i*)^(θ), and *g*_XY_^(*i*)^(*r*) are computed as classical NVT averages with this effective potential. In the basic IBI algorithm, the potential for the next iteration is then found by updating the potential corrections according to
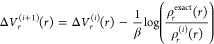
11a
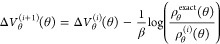
11b
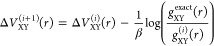
11cwhere β = 1/*k*_B_*T*. The exact distribution functions in these updating formulae are precomputed in a PIMD simulation as described above.

The most expensive part of this algorithm is the calculation of the PIMD distribution functions. The second most expensive part is the calculation of the effective classical distribution functions, ρ_*r*_^(*i*)^(*r*), ρ_θ_^(*i*)^(θ), and *g*_XY_^(*i*)^(*r*), at each IBI iteration. This is facilitated by the recent introduction of variationally optimized^26^ low-variance force estimators^27^ for classical distribution functions. With these new estimators, it is possible to calculate the distribution functions with so little effort that IBI is perfectly feasible, even when many iterations are required for convergence. The effort of the IBI does not even approach that of the PIMD calculation until the number of iterations exceeds the number of path integral beads, which we have not found to happen in any of our calculations.

In the present calculations, we shall follow Althorpe and coworkers^14^ and Kapil and coworkers^22^ in using the q-TIP4P/f water model^28^ to study liquid water and ice. This leads to further simplification in that the intramolecular part of the q-TIP4P/f potential is a sum of the O–H bond length and H–O–H bond angle terms of the same form as Δ*V*_intra_(r) in eq 9. In practice, we find that it is sufficient to assume that each correction, Δ*V*_*r*_^(*i*)^(*r*) and Δ*V*_θ_^(*i*)^(θ), is a polynomial of the same degree as in the original intramolecular potential, which implies that we can simply adjust the parameters in the intramolecular potential rather than update Δ*V*_*r*_^(*i*)^(*r*) and Δ*V*_θ_^(*i*)^(θ) at each IBI iteration.

The corrections to the intermolecular potentials Δ*V*_XY_^(*i*)^(*r*) are more problematic, however. We have found that in order to obtain a convergent sequence of these corrections, it is essential to regularize the intermolecular IBI update, as we shall describe next.

### Regularized IBI

There are heuristic arguments to suggest that the IBI algorithm will always find a solution,^20^ and progress has recently been made toward proving its convergence.^29^ In practice, however, we have found (in agreement with others^21^) that close to convergence, the iterations can start to oscillate and eventually even become unstable. Furthermore, in regions where the radial distribution functions *g*_XY_(*r*) are small, the IBI update can become dominated by statistical errors.

We can overcome both problems by adding a regularization term to the radial distribution functions before performing the IBI update. The new update formula is
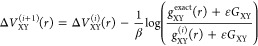
12where ε is a non-negative scalar parameter and *G*_XY_ is the larger of the maximum peak heights in *g*_XY_^exact^(*r*) and *g*_XY_^(*i*)^(*r*). This definition of *G*_XY_ allows us to use the same value of ε for all three radial distribution functions (OO, OH, and HH). The regularization in eq 12 was first proposed by Soper,^20^ but it has (to the best of our knowledge) not been picked up in later applications of IBI. There is no need to regularize the intramolecular correction because only the regions of ρ_*r*_(*r*) and ρ_θ_(θ) with low statistical noise need be used in the adjustment of the intramolecular potential parameters.

**Figure 1:**
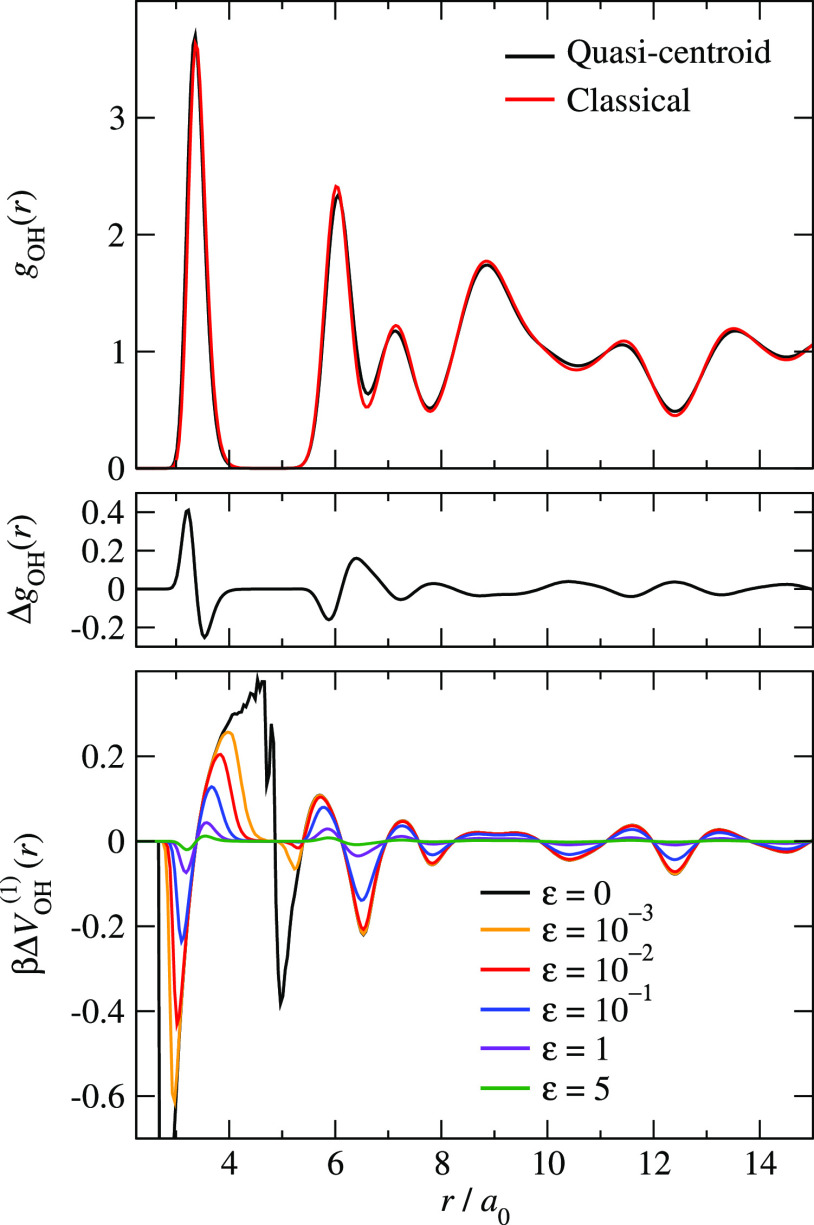
Influence of the regularization parameter ε on the shape of the first IBI update to the intermolecular O–H potential in ice at 150 K. The top panel shows the classical and quasi-centroid radial distribution functions, the middle panel shows the difference between them, and the bottom panel shows the correction potential for different values of ε.

Figure 1 shows the effect of ε on the first update to the O–H intermolecular potential for ice at 150 K. We see that the regularization has the most noticeable effect on regions with little density: in the short-range tail of the distribution and in the well between the first and second peaks. Without regularization, the correction to the potential is clearly noisy in these regions, and it can become very large. The large corrections overshoot the target potential of mean force, leading to wild oscillations in the convergence of the IBI, if it converges at all. Even relatively small values of the regularization parameter are sufficient to remove these instabilities. Furthermore, since each correction to the potential of mean force is now free from statistical errors, which are suppressed by the *ε G*_XY_ terms in eq 12, the final result of the IBI is a potential with smooth forces that is suitable for use in molecular dynamics simulations.

### Quasi-Centroid Molecular Dynamics

Once the IBI has converged, the resulting quasi-centroid potential of mean force *V*_qc_(**r**) can be used to calculate the dipole absorption spectrum from the standard formula
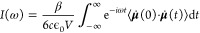
13where the angular brackets denote a canonical ensemble average, the dynamics is purely classical, and **μ̇** is the time derivative of the dipole moment of the system (here liquid water or ice). This is no more difficult than performing a standard classical molecular dynamics simulation of the spectrum on potential *V*_cl_(**r**).

## Application to Liquid Water and Ice

### Computational Details

**Figure 2:**
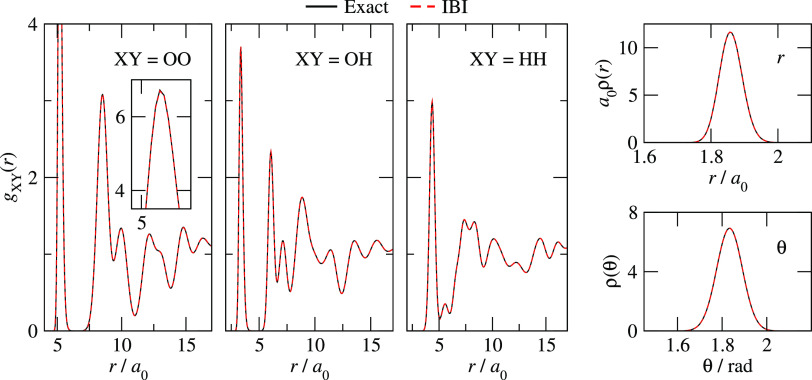
Comparison of exact PIMD and fully converged IBI quasi-centroid distribution functions for hexagonal ice at 150 K.

We used the IBI algorithm described in Sec. 2 to calculate the f-QCMD approximation to the quasi-centroid potential of mean force for q-TIP4P/f water at 300 K and ice at 150 K. All simulations used a time step of 0.25 fs. The liquid water simulation was performed using 216 water molecules in a cubic box with a side length of 35.24 *a*_0_, and the ice simulation with 96 molecules in an orthorhombic box with side lengths of 25.62, 29.58, and 27.89 *a*_0_. Following refs (15) and (17) the PIMD simulations used *P* = 32 beads at 300 K and *P* = 64 beads at 150 K. We performed a total of 30 regularized IBI iterations at both 300 and 150 K, with ε = 1 and ε = 5, respectively. At both temperatures, the quasi-centroid distribution functions obtained from IBI were found to be graphically indistinguishable from the target distribution functions provided by the PIMD simulation. The distribution functions for ice are shown in Figure 2, and those for liquid water are shown in Figure S1 of the Supporting Information.

At this point, we should stress that the agreement between the distribution functions does not guarantee that the potential recovered by the IBI iteration is correct. This has only been shown to be the case in the idealized situation in which IBI is used to recover a pair potential (to within a constant) from a pair distribution function.^30^ As discussed by Evans,^31^ third and higher-order correlation functions are needed to capture higher-order interactions. In what we are doing here, the situation is more complicated because we are not using IBI to recover the potential of mean force itself but rather the (relatively small) difference between it and the underlying classical potential (which does contain higher-order interactions). So, our IBI solution may not be unique, and we should even be concerned that using different values of ε might lead to different results. We have investigated this and found that while varying ε (within the domain of convergence) does lead to subtle differences in the converged quasi-centroid potential of mean force, these differences do not have any observable effect on the vibrational spectrum.

### Liquid Water

**Figure 3:**
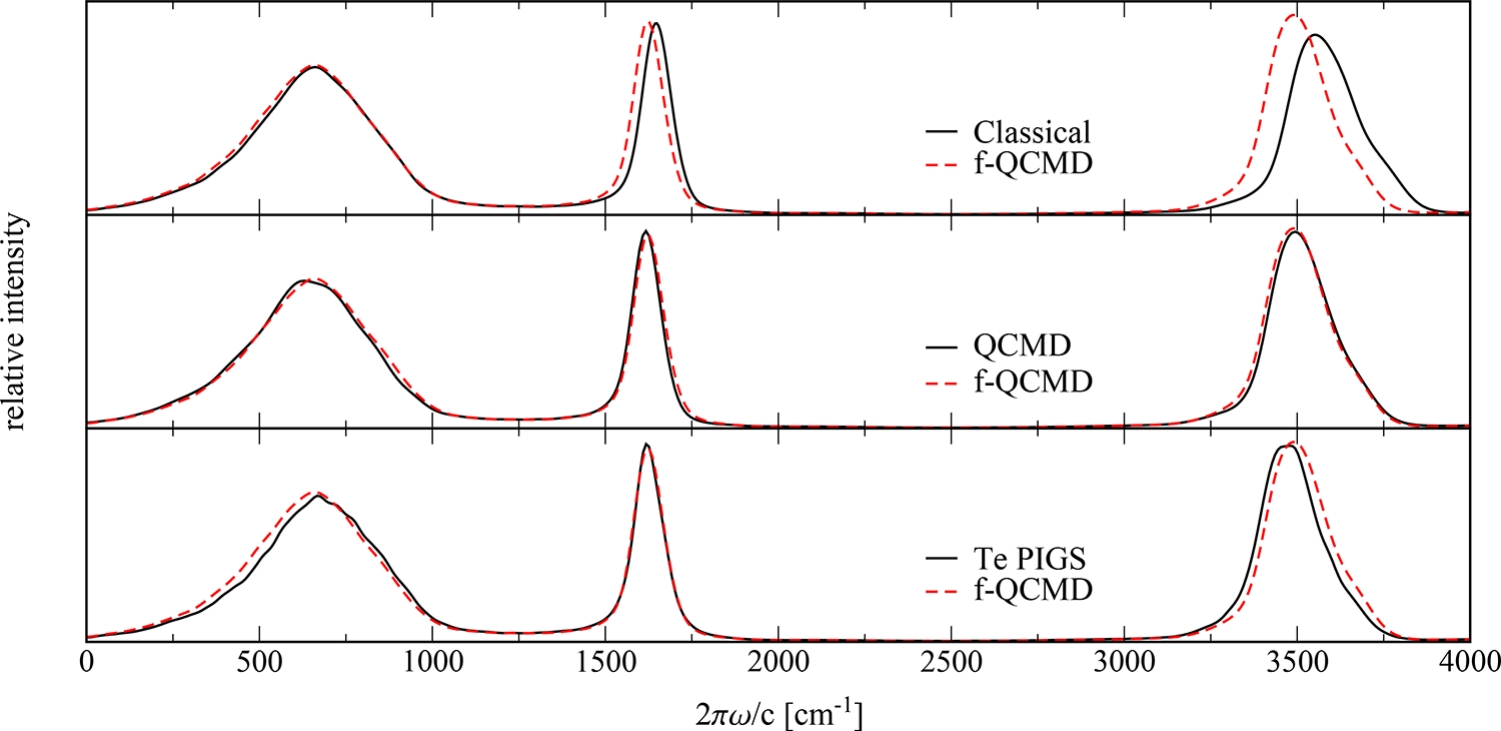
Comparison of classical MD, fast QCMD, adiabatic QCMD, and Te PIGS vibrational spectra of qTIP4P/f water at 300 K.

Figure 3 compares the resulting f-QCMD spectrum for liquid water at 300 K with the spectra obtained from classical molecular dynamics, the adiabatic QCMD method,^15^ and the Te PIGS method.^22^ (The adiabatic QCMD results were obtained using the new torque estimator described in ref (17), and were provided to us by George Trenins). All spectra were computed from the dipole-derivative autocorrelation function in eq 13 damped using a Hann window function with a time constant of 600 fs. Here, we are only considering the fundamental region of the spectrum, which has three major peaks: a librational band at 600 cm^–1^, a bending band at 1600 cm^–1^, and an O–H stretching band at 3500 cm^–1^.

The first thing to note is that the agreement between the f-QCMD spectrum and the adiabatic QCMD spectrum is very good, with f-QCMD accurately reproducing both the line shapes and the frequencies of the adiabatic QCMD peaks. This indicates that the separable approximations to the correction potentials in eqs 9 and 10 are perfectly adequate for liquid water at 300 K. The only differences between the two spectra are a very minor red shift in our stretching peak and equally minor blue shifts in our librational and bending bands. The much larger differences between the f-QCMD spectrum and the classical spectrum are due to nuclear quantum effects, which result in an anharmonic red shift of around 100 cm^–1^ in the O–H stretching band and a smaller red shift in the bending band. The classical and f-QCMD librational bands are essentially the same because nuclear quantum effects are less significant for low-frequency vibrations.

The final panel of Figure 3 compares the f-QCMD spectrum with the Te PIGS spectrum obtained using a centroid potential of mean force calculated at 600 K.^22^ The bending bands of the two spectra are the same, but the Te PIGS librational band is slightly blue-shifted, and the Te PIGS O–H stretching band is slightly red-shifted relative to the f-QCMD spectrum. The blue shift in the librational band is most likely a result of incomplete convergence of the autocorrelation function used to calculate the spectrum, as this is the region of the spectrum that converges most slowly. This is corroborated by the slightly uneven line shape of this peak in the Te PIGS calculation. While the slight red shift in the stretching band might also be due to incomplete convergence, it could possibly be a hint of the CMD curvature problem, which is just starting to appear in the centroid potential of mean force at 600 K.^15^

### Hexagonal Ice

**Figure 4:**
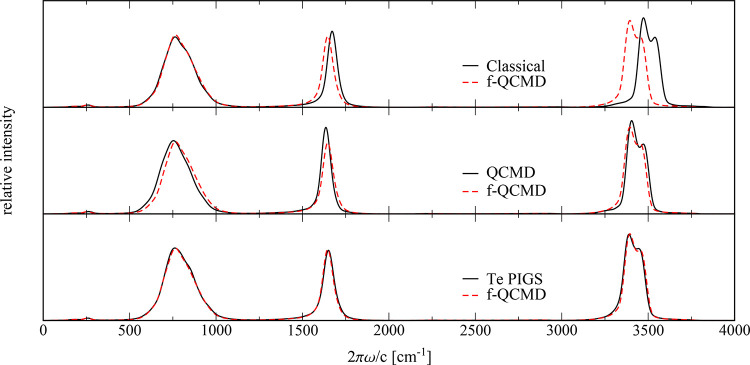
Comparison of classical MD, fast QCMD, adiabatic QCMD, and Te PIGS vibrational spectra of qTIP4P/f ice at 150 K.

Figure 4 shows the vibrational spectra for hexagonal ice at 150 K. These spectra were computed in the same way as the liquid water spectra but using a Hann window function with a time constant of 800 fs. They differ from the liquid water spectra in that all three of the main bands are narrower, there is more structure in the O–H stretching band, and a tiny intermolecular O–O stretching band is now apparent at around 250 cm^–1^.

Comparing the QCMD and f-QCMD spectra in Figure 4, it is clear that the differences in ice at 150 K are more pronounced than they were in water at 300 K. The f-QCMD bending band is less intense, the librational band is slightly blue-shifted, and the stretching band is slightly red-shifted relative to QCMD. The most likely explanation for these differences is that the pairwise approximations to the correction potentials in eqs 9 and 10 are less accurate in ice at 150 K than they were in the water at 300 K. Alternatively, the discrepancy could be caused by a lack of convergence with respect to the separation parameters in the adiabatic QCMD algorithm, or by errors introduced by the approximate torque estimator used in QCMD. These possibilities are suggested by Figures S3 and S4 in the Supporting Information, which show that the quasi-centroid distribution functions obtained from the adiabatic QCMD trajectories of Trenins et al.^17^ do not agree quite so well with our reference PIMD calculations for ice as they do for water. The peak positions in the adiabatic QCMD ice spectrum were found to be converged with respect to the adiabatic separation parameters,^32^ so this is probably not the root of the disagreement with f-QCMD. Regarding the torque estimator, ref (17) shows that both QCMD and CMD predict essentially the same red shifts relative to the classical results in the librational and bending bands. As these regions are not expected to be significantly affected by the curvature problem of CMD, this suggests that the errors in the torque estimator are minor. We thus believe that the QCMD peak positions are unlikely to be affected by either of these potential sources of error, although we do not rule out the possibility that the intensities of the QCMD bands may be slightly incorrect.

Interestingly, the agreement between the Te PIGS and f-QCMD spectra in Figure 4 is nearly perfect. While this is probably somewhat fortuitous,^33^ it is worth speculating about why it might be. The following explanation assumes that the QCMD results are the most accurate in Figure 4 and that the Te PIGS results are better converged for ice than they are for water. We have already argued that our pairwise approximation to the correction potential Δ*V*(**r**) = *V*_qc_(**r**) – *V*_cl_(**r**) is justified at high temperatures by the good agreement between f-QCMD and QCMD for liquid water at 300 K. Since the centroid potential of mean force correction used in Te PIGS is calculated at an even higher temperature (600 K), it too is presumably dominated by pairwise terms. Hence, it is possible that Te PIGS and f-QCMD are both missing the many-body contributions to Δ*V*(**r**) that arise at lower temperatures and lead to the additional red shifts of the librational and bending bands in the QCMD ice spectrum but for different reasons: Te PIGS because its potential of mean force is computed at a high temperature where these contributions are less significant and f-QCMD because it inherently assumes that Δ*V*(**r**) can be written in a pairwise form. Note that since this argument is based on the agreement between f-QCMD and QCMD for water at 300 K (Figure 3) and the agreement between f-QCMD and Te PIGS for ice at 150 K (Figure 4), it would not have been possible to make it without the results of the present calculations.

### Summary

In spite of their differences, it is clear from Figures 3 and 4 that all three centroid methods (QCMD, f-QCMD, and Te PIGS) are in better agreement with one another than they are with classical molecular dynamics and that they provide reasonably consistent predictions of the dominant quantum mechanical effects in the spectra of water and ice (an anharmonic red shift in the intramolecular stretching band and a smaller red shift in the intramolecular bending band, with little change in the line shape of either). This is clear progress compared with the situation a decade or so ago when the best existing path integral methods were giving markedly different results for these spectra that were plagued by artifacts. See, for example, Figure 2 in ref (34) or Figures 3 and 4 in ref (14) for adiabatic CMD^6^ and thermostatted ring polymer molecular dynamics^35^ spectra that can be compared directly with those in our Figures 3 and 4.

One may ask whether these methods not only agree with each other but also whether they agree with the exact result. For a condensed-phase system, it is, of course, not possible to obtain the exact result for comparison. However, for the gas-phase water monomer, both QCMD and f-QCMD were found to give remarkably good agreement with the quantum reference calculations for the fundamental bands.^18^ This can be understood by noting that in the low-temperature limit, centroid molecular dynamics gives the exact 0 → 1 transition frequency for an anharmonic oscillator in one dimension,^11^ and that within the coordinate frame used in QCMD, the normal modes are only weakly coupled. On going to the condensed phase, one notes that from a single monomer perspective, the exact quantum statistics of QCMD means the static effect of the environment is captured exactly. Furthermore, the dynamical effect of the environment is captured self-consistently within a classical framework. One can therefore have reasonable confidence that these methods are converging not only to one another but also on an accurate description of the fundamental bands of the vibrational spectrum.

## Concluding Remarks

Modern centroid methods are finally reaching a consensus regarding the impact of nuclear quantum effects on the vibrational spectra of water and ice. For the q-TIP4P/f water model studied here, they lead to an anharmonic red shift of around 100 cm^–1^ in the intramolecular O–H stretching band and a smaller red shift in the intramolecular H–O–H bending band but very little change in the line shape of either band (see Figures 3 and 4). Recent Te PIGS calculations demonstrate that this remains largely true for calculations using *ab initio* DFT potential energy surfaces, albeit with larger changes to the intensities and the line shapes of the bands in ice.^36^

There will be many situations in which it is reasonable to ignore the relatively small quantum effects that we are discussing here and use classical molecular dynamics to simulate the vibrational spectrum. However, there will be other situations where the goal is to understand anharmonic red and blue shifts in vibrational spectra and how they change with temperature and isotopic substitution, which are questions that classical molecular dynamics is incapable of answering.^37^ In these situations, we feel fairly confident on the basis of the present results and previous validation studies for gas-phase systems^15,16,18,22^ (for which exact quantum mechanical benchmark results are available for comparison) that methods like QCMD, f-QCMD, and Te PIGS will provide the right answer.

Given this, we are now in a position to apply these methods to more interesting problems, and we have, in fact, already completed two such studies using f-QCMD.^38^ The present quasi-centroid potential of mean force for liquid water has been used to shed light on claims that nuclear quantum effects may broaden vibrational polariton bands,^38,39^ and to explore the impact of nuclear quantum effects on the thermal conductivity of liquid water^39^ using the method introduced in ref (40). For most future applications, we would suggest that the more recent Te PIGS method^22^ should be the preferred approach. We have argued in Section 3.3 that Te PIGS is not as accurate as adiabatic QCMD, but it is significantly simpler to implement, and it appears to give comparable accuracy to the pairwise approximation to Δ*V*(**r**) that is made in f-QCMD. It should, therefore, be perfectly adequate for studying anharmonic effects in many interesting gas and condensed-phase systems.

One final comment is that we have focused exclusively on the fundamental bands. Centroid methods are less accurate for overtone and combination bands, most notably failing to capture their quantum mechanical intensity enhancement.^41,42^ Within the framework of Matsubara dynamics,^43^ this has been shown to be a result of decoupling the centroid mode from the fluctuation modes of the path integral.^42^ An alternative method based on coupling to an effective thermal bath that mimics the so-called “Matsubara heating” effect has been proposed,^44^ as have postprocessing corrections.^42^ Hence, this is clearly an area in which there is still room for improvement in the methods we have considered here.

## Data Availability

The data to support this study are available in the body of the paper and in the Supporting Information; code to perform the iterative Boltzmann inversion is available upon request.

## Supporting Information Available

Figures showing the convergence of the iterative Boltzmann inversion to the exact distribution functions and the difference between the exact and adiabatic QCMD distribution functions (PDF)
