# Supplementary material for: Fast Quasi-Centroid Molecular Dynamics for Water and Ice
Source: J Phys Chem B. 2023 Oct 13;127(42):9172–80. doi: 10.1021/acs.jpcb.3c05028 (PMC10614180; doi:10.1021/acs.jpcb.3c05028)
Supplement: Supplementary file 1 — jp3c05028_si_001.pdf [file jp3c05028_si_001.pdf]

Supporting Information: Fast Quasi-Centroid Molecular Dynamics for Water and Ice

Joseph E. Lawrence,[*,†] Annina Z. Lieberherr,[‡] Theo Fletcher,[‡] and David E. Manolopoulos[‡]

†*Laboratory of Physical Chemistry, ETH Zürich, 8093 Zürich, Switzerland*

‡ *Physical and Theoretical Chemistry Laboratory, Department of Chemistry, University of Oxford, South Parks Road, Oxford OX1 3QZ, United Kingdom*

E-mail: joseph.lawrence@phys.chem.ethz.ch

Convergence of the IBI algorithm

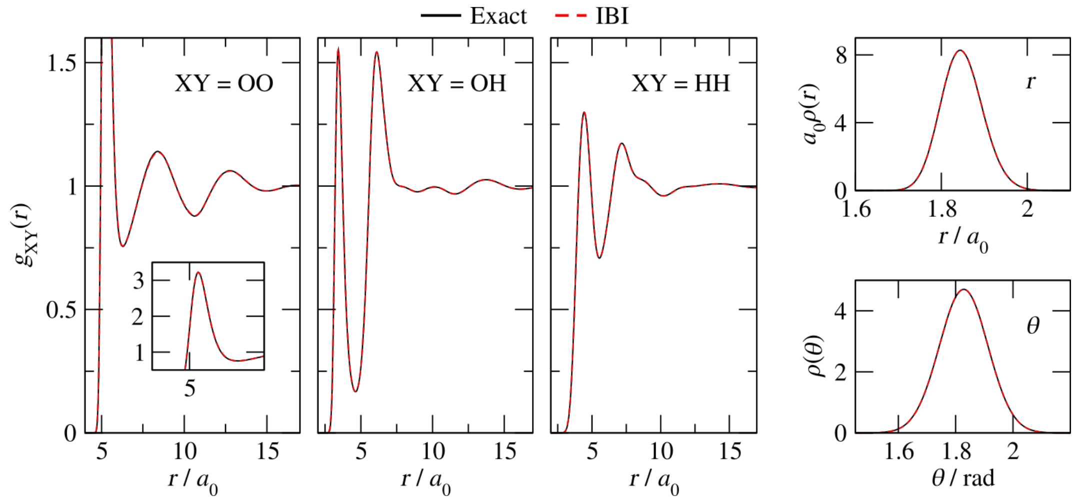

Figure S1: Comparison of distribution functions in the final IBI iteration to the exact distribution functions from a PIMD calculation for liquid water at 300 K. (This is the same as Fig. 2 in the paper but we have included it here to facilitate the comparison with the ice results in Fig. S2)

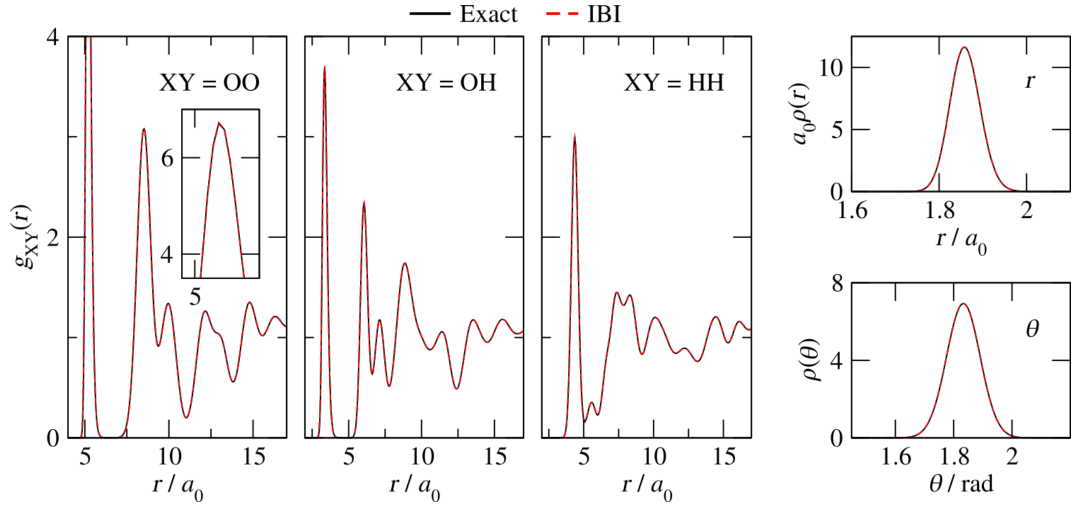

Figure S2: As figure S1, but for hexagonal ice at 150 K.

Quasi-centroid distribution functions

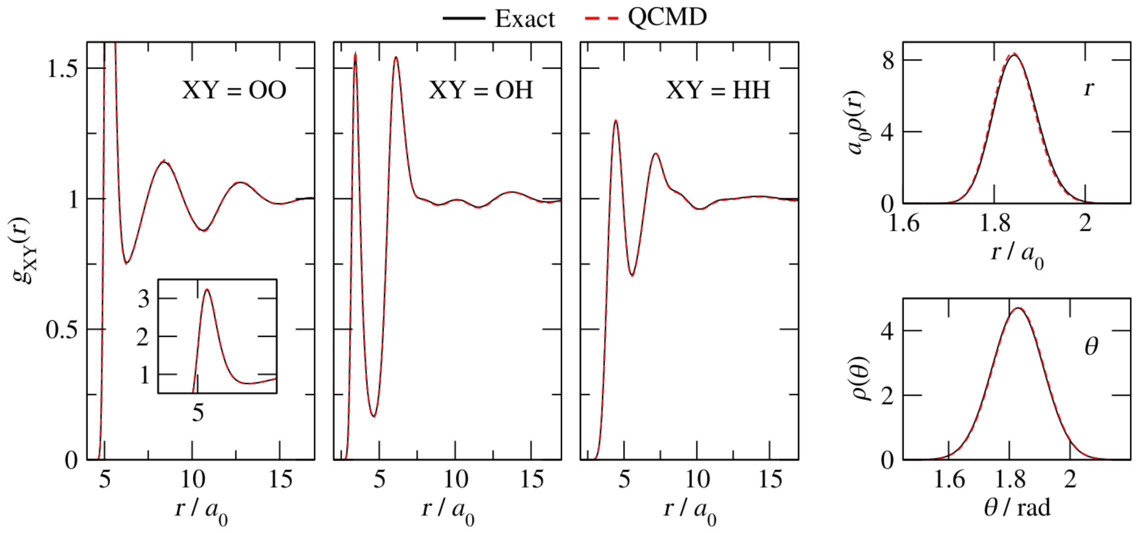

Figure S3: Quasi-centroid distribution functions averaged over a PIMD trajectory (black) and an adiabatic QCMD trajectory (red, dashed) for liquid water at 300 K.

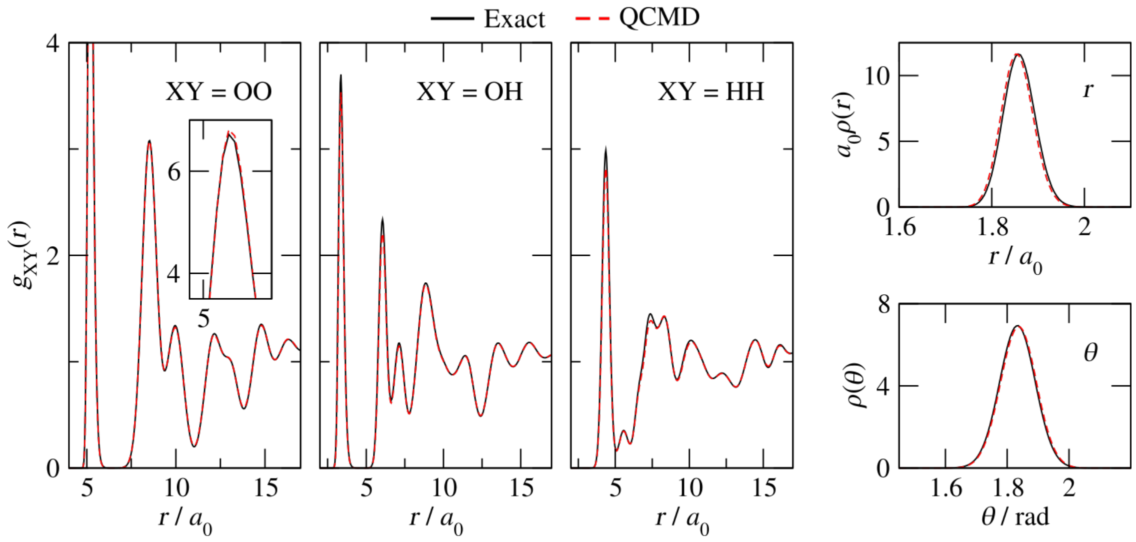

Figure S4: As Fig. S3, but for hexagonal ice at 150 K.
